# The promise of digital healthcare technologies

**DOI:** 10.3389/fpubh.2023.1196596

**Published:** 2023-09-26

**Authors:** Andy Wai Kan Yeung, Ali Torkamani, Atul J. Butte, Benjamin S. Glicksberg, Björn Schuller, Blanca Rodriguez, Daniel S. W. Ting, David Bates, Eva Schaden, Hanchuan Peng, Harald Willschke, Jeroen van der Laak, Josip Car, Kazem Rahimi, Leo Anthony Celi, Maciej Banach, Maria Kletecka-Pulker, Oliver Kimberger, Roland Eils, Sheikh Mohammed Shariful Islam, Stephen T. Wong, Tien Yin Wong, Wei Gao, Søren Brunak, Atanas G. Atanasov

**Affiliations:** ^1^Oral and Maxillofacial Radiology, Applied Oral Sciences and Community Dental Care, Faculty of Dentistry, University of Hong Kong, Hong Kong, China; ^2^Ludwig Boltzmann Institute Digital Health and Patient Safety, Medical University of Vienna, Vienna, Austria; ^3^Department of Integrative Structural and Computational Biology, Scripps Research Translational Institute, La Jolla, CA, United States; ^4^Bakar Computational Health Sciences Institute, University of California, San Francisco, San Francisco, CA, United States; ^5^Department of Pediatrics, University of California, San Francisco, San Francisco, CA, United States; ^6^Department of Genetics and Genomic Sciences, Icahn School of Medicine at Mount Sinai, New York, NY, United States; ^7^Hasso Plattner Institute for Digital Health at Mount Sinai, Icahn School of Medicine at Mount Sinai, New York, NY, United States; ^8^Department of Computing, Imperial College London, London, United Kingdom; ^9^Chair of Embedded Intelligence for Health Care and Wellbeing, University of Augsburg, Augsburg, Germany; ^10^Department of Computer Science, University of Oxford, Oxford, United Kingdom; ^11^Singapore National Eye Center, Singapore Eye Research Institute, Singapore, Singapore; ^12^Duke-NUS Medical School, National University of Singapore, Singapore, Singapore; ^13^Department of General Internal Medicine, Brigham and Women’s Hospital, Harvard Medical School, Boston, MA, United States; ^14^Department of Anaesthesia, Intensive Care Medicine and Pain Medicine, Medical University of Vienna, Vienna, Austria; ^15^Institute for Brain and Intelligence, Southeast University, Nanjing, China; ^16^Department of Pathology, Radboud University Medical Center, Nijmegen, Netherlands; ^17^Primary Care and Public Health, School of Public Health, Imperial College London, London, United Kingdom; ^18^Centre for Population Health Sciences, LKC Medicine, Nanyang Technological University, Singapore, Singapore; ^19^Deep Medicine Nuffield Department of Women’s and Reproductive Health, University of Oxford, Oxford, United Kingdom; ^20^Institute for Medical Engineering and Science, Massachusetts Institute of Technology, Cambridge, MA, United States; ^21^Department of Medicine, Beth Israel Deaconess Medical Center, Boston, MA, United States; ^22^Department of Biostatistics, Harvard T.H. Chan School of Public Health, Boston, MA, United States; ^23^Department of Preventive Cardiology and Lipidology, Medical University of Lodz (MUL), Lodz, Poland; ^24^Department of Cardiology and Adult Congenital Heart Diseases, Polish Mother’s Memorial Hospital Research Institute (PMMHRI), Lodz, Poland; ^25^Institute for Ethics and Law in Medicine, University of Vienna, Vienna, Austria; ^26^Digital Health Center, Berlin Institute of Health (BIH), Charité – Universitätsmedizin Berlin, Berlin, Germany; ^27^Institute for Physical Activity and Nutrition, Deakin University, Geelong, VIC, Australia; ^28^Department of Systems Medicine and Bioengineering, Houston Methodist Cancer Center, T. T. and W. F. Chao Center for BRAIN, Houston Methodist Academic Institute, Houston Methodist Hospital, Houston, TX, United States; ^29^Departments of Radiology, Pathology and Laboratory Medicine and Brain and Mind Research Institute, Weill Cornell Medicine, New York, NY, United States; ^30^Tsinghua Medicine, Tsinghua University, Beijing, China; ^31^Andrew and Peggy Cherng Department of Medical Engineering, California Institute of Technology, Pasadena, CA, United States; ^32^Novo Nordisk Foundation Center for Protein Research, Faculty of Health and Medical Sciences, University of Copenhagen, Copenhagen, Denmark; ^33^Institute of Genetics and Animal Biotechnology of the Polish Academy of Sciences, Jastrzebiec, Poland

**Keywords:** digital health, biosensors, bioinformatics, telehealth, precision medicine

## Abstract

Digital health technologies have been in use for many years in a wide spectrum of healthcare scenarios. This narrative review outlines the current use and the future strategies and significance of digital health technologies in modern healthcare applications. It covers the current state of the scientific field (delineating major strengths, limitations, and applications) and envisions the future impact of relevant emerging key technologies. Furthermore, we attempt to provide recommendations for innovative approaches that would accelerate and benefit the research, translation and utilization of digital health technologies.

## 1. Digital health technologies: a snapshot

### 1.1. General significance of digital health technologies through history until today

According to the World Health Organization (WHO), digital health technologies can be defined as: *“the field of knowledge and practice associated with the development and use of digital technologies to improve health… Digital health expands the concept of eHealth to include digital consumers, with a wider range of smart and connected devices. It also encompasses other uses of digital technologies for health such as the Internet of Things (IoT), advanced computing, big data analytics, artificial intelligence including machine learning, and robotics”* (1). Importantly, in the context of digital health technologies, several terms such as eHealth (electronic health), telemedicine, and mHealth (mobile health) have been widely used, unfortunately on some occasions with overlapping meaning, underlining the necessity of using more precise scientific language reflecting the subtle differences between such relevant terms (2). Along this line, “digital health” has been coined to represent the broadest term covering the application of digital technologies in the context of health, and while being rooted in electronic health, this term also encompasses other adjacent areas such as “big data” applications, genomics, and artificial intelligence. Further, eHealth is often referred to as the use of information and communications technology in support of health, mHealth is viewed as a branch of eHealth that refers to the use of wireless mobile technologies for public health (gaining particular momentum with the wide adoption of smartphones and respective apps), and telemedicine is a term reflecting the use of electronic communications and information technologies for remote provision of health care services (2).

Telemedicine, for example, has been a contemporary recurring discussion topic in the scientific, government and healthcare community, especially when the majority of the global population has experienced various scales of community lockdowns, home quarantines, and reduced availability of medical services during the COVID-19 global pandemic (3). The COVID-19 pandemic that began in early 2020 accelerated the expansion and implementation of existing and novel digital health technologies through increasing funding, fast-track policy approvals, enhanced governmental priorities, new private-public partnerships, and the pooling and planning and design of various collaborative research (4). On hindsight, the utilization of telemedicine should have started as soon as the phone came into use by physicians. Since its invention in 1876, the telephone has been used as a tool for delivering healthcare: Alexander Graham Bell’s first recorded telephone call was for medical help after he spilt sulphuric acid on himself (5, 6).

In the late 20^th^ century when the healthcare industry began to first embrace computerization and incorporate information technology, the initial intentions were focused on streamlining procedures to reduce manually introduced errors in the workflow. The impact of medical errors can be minimized by preventing erroneous entries and by mitigating the risk of adverse events, facilitating a more prompt response after an adverse event has occurred, and tracking and providing feedback about any adverse event (7). For example, a computer-based decision support systems could identify interactions between different drugs taken by a patient and prevent adverse drug events (7–9). Meanwhile, a physician computer order entry system (CPOE) reduced 55% of the non-intercepted serious medication errors in a hospital located in Boston, the United States (10). Along the chain of steps in the workflow from diagnosis to medication, digital health technologies such as electronic documentation, bar coding, and robots/automated dispensing devices can be helpful in reducing errors (11, 12). More recently, research has focused on digital health technologies on three main aspects: data storage, management, and transmission; clinical decision support; and telemedicine (13). However, it is not clearly evident that these have led to substantially improved clinical care or improved cost-effectiveness of healthcare services.

Currently, the spectrum of digital health technologies has expanded and includes not only telemedicine (concept that was developed before the time of digital technologies, but was markedly reshaped from the latter), but analysis and utilization of big data, comprehensive health record digitization, IoT, wireless and mobile technology/5G, blockchain, artificial intelligence and machine learning (AI/ML) including deep learning, and wearable monitors (biosensors). The increasing accessibility of cloud computing and cloud storage may facilitate more complex diagnostic procedures via telemedicine, such as bioimage analysis that requires computational power not locally available (14–17). The near ubiquitous penetration of mobile phone to vast populations across the globe will likely play an increasing role in digital health technologies, with this upcoming research field being coined as mHealth (18, 19). Particularly with the big data from electronic patient records (or real-world data (20)) that forms a digital knowledge base, AI analyses can be performed to aid diagnosis and treatment selection, resulting in an improved clinical decision support (21) and image-based medical diagnosis (22).

Compared with traditional healthcare, digital healthcare can potentially be more precise, less error-prone, and more efficient [Table 1, based on Meskó et al. (23)]. With consideration of the listed developments, the present work aims to give perspective of the promise of digital health technologies in healthcare.

**Table 1:** Differences between traditional and digital healthcare.

Traditional healthcare	Digital healthcare
Direct patient-physician relationships	Patient-machine-physician interface
Standardized care based on physician experience and standard clinical workflow: Symptoms, clinical signs, ancillary medical tests, diagnosis, and treatment plan	Individualized care, precision medicine, with non-traditional workflow: Mass screening, early preclinical or asymptomatic diagnosis, diagnosis based on probability, predictive technology, and decision support for physicians
Point of care delivery or examination is at the clinic or lab	Point of care delivery or examination may vary as long as patient is present
Data owned by the institutions/hospitals	Data owned and shared by multiple stakeholders, including the patient
Physician as the central player who makes diagnosis, and prescribes treatment plan	Physician as a consultant, guide or collaborator with the patient’s active contribution in the decision making

The table is based on Meskó et al. (23), with adaptations from the authors.

### 1.2. Challenges associated with digital health technologies

There are major challenges in the implementation of new technologies, particularly disruptive technologies, in an established industry such as healthcare. From the patient perspective, the first obvious challenge with digital health technologies is the inability to use the technology or mobile phones due to low digital health literacy or low access to technology, especially for the older adults and people with lower digital literacy (24). Second, poor app design may hinder the implementation or growth of digital health technologies, such as being one of the barriers to adopting telemedicine besides staff technology level, resistance to change, cost, and patient age and education level (25). Without clear advantage and ease of use, physicians may not have the incentive to implement the technologies or ask patients to use them.

Poor App design may only make the usage less efficient, but a more severe issue is the lack of rigorous regulations for assurance of quality/effectiveness or insufficient validation of clinical effectiveness. This may seriously tarnish the general impression of digital health technologies by the public/society. For instance, it was found that mental health apps, totally downloaded more than 2 million times, provided non-existent or inaccurate suicide crisis helpline phone numbers, with only 5 out of 69 depression and suicide prevention apps offering all six evidence-based suicide prevention strategies (26). While the introduction of new medication into clinical practice requires rigorous evaluation and regulation and involves head-to-head studies, it seems that health apps can be introduced into the market with less quality assurance. This implied further needs for improvement at the policy level. For example, the performance of a dermatology app was tested with biopsy-proven melanoma pictures, and it was found that the app could only label 11% of the pictures as high risk and another 88% as medium risk (27). This situation seemed to be gradually improving as technology has become more matured. A more recent study that evaluated 8 symptom assessment apps on their “*breadth of condition coverage, accuracy of suggested conditions and appropriateness of urgency advice*” has found that some apps had comparable performance with general practitioners (GPs) in these aspects, but still, none could outperform GPs (28). Unfortunately, the mainstream reviews of mHealth apps have been hugely based on personal experiences, with few evidence-based, unbiased evaluations of clinical performance and data security (29). As such, the accuracy or usefulness of the apps should be further verified before their release for clinical use (30). One crucial aspect that should perhaps be highlighted was that there seemed to be no study of the clinical risks and benefits of the apps involving real-world consumer use (31).

Among other frequently recognized challenges such as insufficient technology support, high cost, privacy and security concerns, and compatibility with digital solutions established at hospital/ambulatory systems, data ownership uncertainty stands out as a particularly important present and future issue. There is always a “fight” between big companies owning the data and charging for every analysis and access, versus patients/healthcare professionals owning it and retaining their rights to use it in whatever ways are deemed to yield better care. In this context, a recent literature review has summarized the following points (32): (1) there was wide concern about the security of mHealth data storage and transmission; (2) aggregated data previously considered “de-identified” could actually be re-identifiable; (3) there might be a lack of consumer-informed consent due to the absence of a privacy policy or the respective policy text being too complex and lengthy; and (4) improved access control should be advocated. Some of the ethical considerations are specifically elaborated below:

#### 1.2.1. Privacy and security

Digital health technologies collect and store large amounts of personal health information. There is a risk that this information could be accessed or breached by unauthorized personnel, leading to privacy issues, potential identity theft, and other forms of damage. It is critical to ensure that digital health technologies comply with data privacy regulations, have robust security measures, and provide patients with adequate control over their data privacy settings.

#### 1.2.2. Informed consent

Patients should have the right to be informed and to participate in their healthcare decisions. The use of digital health technologies should not omit or dismiss the importance of obtaining informed consent from the patients, especially if they are unaware of what data would be collected and how the data would be distributed. Also, it is important to allow patients to opt-out of data collection and sharing in an explicit and straightforward manner.

#### 1.2.3. Algorithmic bias

Digital health technologies rely on algorithms to analyze health data and provide recommendations/decisions regarding patient care. If the algorithms are biased, unfair or discriminatory treatment of patients might happen. Hence, it is crucial to validate the algorithms used in digital health technologies to ensure they are unbiased and do not perpetuate existing inequalities before releasing them into the market.

#### 1.2.4. Equity and access

Digital health technologies have the potential to improve healthcare access and equity by providing remote care. However, patients without access to the technology or digital literacy skills will still be excluded. Healthcare providers should ensure that digital health technologies are accessible to all patients, regardless of their socioeconomic status or location.

#### 1.2.5. Professional integrity

The use of digital health technologies may affect healthcare professionals’ professional integrity, as they may rely on technology to make decisions rather than their clinical judgment. Healthcare professionals should be adequately trained in the use of digital health technologies and they should be reminded of their responsibility and liability in providing healthcare.

There are also challenges about the existence of multiple competing technologies in the same area that are often not compatible, and thus data cannot be easily interchanged or transferred between competing platforms (interoperability issue). Data heterogeneity also creates difficulties for the analysis and the interpretation. This was the case with the contact-tracing apps and electronic vaccination records developed during the COVID-19 pandemic: the so-called “format wars” (33). This issue particularly affects more complex multimedia data types including patient videos, audios, digital pathology, IoT, social media, and further. For example, the analysis of neuroimaging data by neuroscientists often begins with data conversion from the Digital Imaging and Communications in Medicine (DICOM) format to the Neuroimaging Informatics Technology Initiative (NIfTI) format, which itself might require some expertise (34). It is because the DICOM specification is complex and allows for variability among different manufacturers to embed customized data into the odd column of DICOM headers (35). Therefore, some researchers advocated that any shared data observe the principles of being findable, accessible, interoperable, and reusable (FAIR) (36). A related problem is that if accurate linkability between data types at the level of individuals could be applied. Not all countries have ubiquitously used personal identification numbers that in principle can make data types linkable even if formatting issues may make linking cumbersome. In the Nordic countries for example, as well as many other places, health data can be linked to socio-economic data unproblematically as the same identification number is used across private organizations, state agencies, municipalities and other data owners. In Denmark the national identification number was for example implemented as early as 1968, making data linkable way back in time (37, 38). To manipulate such a large amount of personal data, secured cloud computing and storage seemed to be the preferred choice.

With regard to the costs involved in implementing digital health technologies, sometimes a lack of cost-effectiveness was established. A recent systematic review (39) on cost-effectiveness studies of digital health technologies found that 2 out of 17 studies on video-conferencing systems reported a lack of cost-effectiveness, which could be attributed to reasons such as upfront training costs and resource-intensive intervention (40).

Meanwhile, the use of digital health technologies might not always improve clinical care. For instance, digital health technologies could not reduce adverse outcomes for pregnant women with gestational diabetes during delivery, such as pre-eclampsia/eclampsia or the need for use of medication (41).

Challenges associated with the application of artificial intelligence/machine learning (AI/ML) are more specific, such as explainability, trustability, fairness, and personalization. For instance, a survey found that patients preferred to have an AI acting as a physician assistant rather than the main physician, implying that there were trust issues to be overcome (42). Meanwhile, the type of training data should also determine the application of the trained model, such as models based on observational data would better refine existing practices instead of discovering new treatment options (43).

A major limitation of many digital health technologies is that they require significant up-front investments, including the purchase of expensive equipment or systems. Thus, these technologies cannot be readily afforded by poorer countries or communities, rendering them not applicable to a global scale. A recent systematic review summarized that the major barriers in poorer countries included infrastructure, equipment, internet, electricity and the digital health technologies themselves, and they could be considered in three levels: project design and implementation factors; factors within the organizational settings; and factors in the broader community environment (44). Key challenges are summarized into technical and non-technical categories and approaches to overcome them are also listed in Table 2.

**Table 2:** Challenges and recommendations to overcome them.

Challenges	Possible approaches to overcome
Technical	
Data structure and heterogeneity (interoperability)	Unify data format, security and sharing requirements
Digital technology infrastructure	Cloud computing and storage; use of blockchain for secured and decentralized data storage and transport
Non-technical (4 Ps)	
Patient (lack of acceptance, privacy issue, lack of motivation, fear of technology, etc.)	More “how to use” quick guides and ready-to-help staff; more patient involvement in the design; more support to caregivers; encourage promotion from the patient’s attending physicians
Physician (resistance, lack of incentives, fear of losing jobs, changing roles, etc.)	System overhaul and accredited points for continuous professional development schemes; establish clarity in regulation and standardization
Public/society (ethics, acceptance, public education etc.)	Promotional campaigns led by celebrities; evaluate and demonstrate evidence of cost-effectiveness
Policy (ethics, financial, regulatory, especially in less resourceful countries)	Lobbying and public-private partnerships; establish clear legal framework regarding reimbursement schemes and data transparency; provide subsidy to cover high start-up costs or incentivize the use

The table is based on Ambrosino et al. (45), Frederix et al. (46); Shimbo et al. (47); Bhyat et al. (48); Naik et al. (49); Murthy et al. (50).

### 1.3. Potential of digital health technologies from the patient perspective

Despite the multiple challenges encountered in inventing and implementing digital health technologies, there are simultaneously significant potential associated with the application of digital health technologies. One major direction of high promise is the facilitation of personalized medicine. Personalized medicine can be defined as “*tailored disease prevention and treatment for individual variability (*e.g.*, genetic and lifestyle differences among patients) … [and its goal is] to match the right treatments at the right dosages for each individual patient at the right time*” (51). It requires a precise analysis of a patient’s health parameters such as vital signs, blood test results, bioimage interpretations, and more. Complex decision models should be built on established large patient databases. Promising results have been for example reported from studies with animal and human data that used calibrated populations of models to predict and explain intersubject variability in cardiac cellular electrophysiology and atrial electrophysiology (52, 53). By participating in personalized omics profiling projects, patients could also be encouraged to implement diet and exercise changes, with the collected data being used to build prediction models to predict personalized physiological responses such as insulin resistance (54). Along the same line, combining existing data from an electronic patient record system together with genomic data could analyze the fine-scale population structure that impacted genetic risk predictions (55).

Another untapped potential of importance is cognitive automation using virtual Avatar doctors to alleviate the shortage of medical specialists in underserved regions and to enable efficient access to care. Currently, it is possible to set up and operate an augmented virtual doctor office through online multimedia platforms such as Second Life (56). With non-invasive sensors and deep neural networks, AI could build a virtual doctor that was able to autonomously interact with a patient via speech recognition and speech synthesis system (57). Such technology is particularly beneficial to remote and rural areas, where primary healthcare is usually very limited due to low population density. As a proof-of-concept, the system could predict type 2 diabetes mellitus.

Digital health technologies could also facilitate access to health services, more direct communication with the healthcare provider, and full access to information storage and sharing to enable better follow-up and clinical decision making (58), as well as promotion of patient empowerment, better patient adherence and compliance, circumventing geographical barriers (Table 3). One hypothetical advantage of a virtual doctor is that it can improve access to healthcare by patients with limited mobility, such as patients with physical disabilities and frail older adults (60). However, it is unclear if they already had the experience or capability to use the technology of a virtual doctor, or if there could be caretakers readily available to teach them or use the technology with them together.

**Table 3:** Summary of the role of digital health technologies in promoting patient engagement and empowerment.

Role	Description
Access to health information	Patients can have access to their health information, including medical records, test results, and self-management tools, *via* digital health technologies. This information empowers patients to make relevant informed decisions more actively together with their healthcare providers.
Improved communication	Digital health technologies facilitate communication between patients and healthcare providers, enabling patients to ask questions, provide feedback, and receive advice and guidance apart from face-to-face consultation sessions. This increased communication may encourage patients to be more aware of their own daily health condition, and have greater satisfaction.
Personalized care	Healthcare providers may deliver personalized care with tailored treatment plans according to the health metrics collected *via* digital health technologies such as wearables. The personalized approach may improve patient engagement and adherence, and increase treatment efficacy.
Remote monitoring	Wearables and remote monitoring systems enable patients to monitor their health at home and share data with their healthcare providers. Healthcare providers can therefore detect and address health issues more pre-emptively, leading to better prognosis and reduced treatment costs.
Self-management	Digital health technologies are tools and resources for patients to manage their health more independently, such as medication reminders, exercise trackers, and nutrition apps. They can increase patient engagement and self-efficacy, leading to improved health outcomes.

The table is based on Lupton (59).

### 1.4. Representative success stories involving digital health technologies

As with the adoption of new technologies, successful stories provide deep insights. One common digital health technology is the bar code technology, which is now frequently implemented in pharmacy. Drug dispensing in a clinical or hospital setting involves many steps that may go wrong especially in a hospital setting, where staff members need to dispense drugs to multiple patients on a regular and timely basis, which may further promote errors to occur. The use of bar codes may insert verification steps in the chain of workflow to ensure that errors will not accumulate and pass to the subsequent steps. For instance, studies at a hospital pharmacy of a 735-bed tertiary care academic medical center found that, when staff were required to scan all doses of medications during dispensing, the incidence of dispensing errors had a significant 93–96% relative reduction, compared to only visual inspection on retrieval (61); on a related note, the rate of potential adverse drug events (errors determined to be potentially harmful to patients) significantly dropped from 3.1 to 1.6% (62). Meanwhile, the traditional rectangular-shaped bar code has been evolving into the square-shaped Quick Response code (QR code) that can be scanned by mobile phones. The QR code has been used by many digital contact-tracing apps during the COVID-19 pandemic (63).

Another common digital health technology that is already proudly used by consumers is the wearable sensor, with a notable example being the Fitbit family of smartwatches. A study reported that steps, heart rate, energy expenditure, and sleep data collected by Fitbit could detect adults at a high risk of depression with around 80% accuracy, sensitivity, and specificity (64). Another study reported that Fitbit could reliably detect sleep–wake states and sleep stage composition relative to polysomnography, particularly in the estimation of rapid-eye-movement (REM) sleep but not N3 sleep (“deep sleep”) (65). Another use of wearables is tag-based real-time locating system (RTLS), which was used for contact tracing in hospital settings during the COVID-19 pandemic (66).

During the COVID-19 pandemic, digital health technologies have gained unprecedented importance as they could help in monitoring, surveillance, detection and prevention of COVID-19 directly and indirectly (67). The analysis of clinical data of COVID-19 patients with AI/federated learning could effectively predict their clinical outcomes such as the necessity to use mechanical ventilation or death at 24 h (68). However, when we look back retrospectively, it should be noted that many prediction model studies were poorly reported with high risk of bias (e.g., potential inclusion of mislabeled data or data from unknown sources) such that their predictive performances might be over-optimistic (69) or of limited potential clinical use (70).

These examples of successful implementation of digital health technologies are summarized in Table 4.

**Table 4:** Examples of successful implementation of digital health technologies.

Study	Healthcare setting	Targeted outcome	Benefit
Poon et al. (61)	A 735-bed tertiary care academic medical center	Drug dispensing error	Scan all doses of medications during dispensing could ↓ 93–96% error relative to visual check
Poon et al. (62)	A 735-bed tertiary care academic medical center	Potential adverse drug events	The use of bar-code system caused adverse event rate to drop from 3.1 to 1.6%
Huang et al. (66)	A COVID-19 screening and treatment center	Contract tracing with COVID-19 patients	The use of RTLS tags had higher sensitivity than smartphone contact tracing app (95.3% vs. 6.5%)
Dayan et al. (68)	20 institutions/hospitals that screened for COVID-19 patients	Oxygen requirements of symptomatic patients with COVID-19	Effectively predicted the clinical outcomes, e.g., the necessity to use mechanical ventilation or death at 24 h

## 2. Digital health technologies: an outlook

### 2.1. Recent technological and scientific developments expected to impact digital health technologies

In the early 2010s, mHealth technologies were anticipated to transform healthcare in the foreseeable future (71). However, the maturation of AI/ML should also not be overlooked. Even more recently, deep learning systems can be utilized for disease detection, such as detecting diabetic retinopathy from retinal images (72) or papilledema from ocular fundus photos (73). Deep learning is also popularly tested for histopathology (74). Under a competition setting, the best AI algorithms developed could detect and grade prostate cancer on biopsy images, with an agreement reaching 0.86 with expert uropathologists (75). In another competition, a deep learning algorithm even outperformed a panel of 11 pathologists in detecting lymph node metastases on tissue section images from women with breast cancer (76). Besides disease detection, AI also showed more reliable treatment strategies for sepsis in intensive care (77). In addition to a single disease entity focus, AI/ML can also be applied to preventive medicine with supplied big data consisted of longitudinal multi-omics data [such as genomics (78)], clinical test results and biomarker analyses acquired in a large cohort (79). Generative deep learning systems may also be used to predict how a drug will impact omics data at the individual patient level, making it feasible to make upfront thought experiments on drug alternatives rather than testing them on a patient sequentially as it normally is done (80).

One of the more recently developed applications that continue rapidly developing are the wearable sensors. Currently, even nanomaterial-enabled wearable sensors have been developed to record various signals belonging to a patient, such as electrophysiological signals (electrocardiography and electromyography), skin temperature, body joint movements, electrochemistry of sweat; or signals belonging to her/his surroundings, such as environmental humidity, ultraviolet level, and visibility, and such sensors have a vast potential to yield individualized health-related data (81).

Important non-wearable sensors include the radio-frequency identification (RFID) systems that are also commonly used in hospital settings to track the location of volunteering staff, patient beds, wheelchairs, and expensive equipment such as special radiology scopes (82). The active RFID tags, also known as the “beacons,” are used to track the real-time location of staff and assets; whereas the passive RFID tags are used to identify patients and facilitate user access to patient data (82).

Gamification is another digital health-related application that can improve patient compliance. Digital technologies, such as virtual reality, can be incorporated into serious games which could bring about significant improvements in attention and memory functions during neuropsychological rehabilitation of stroke patients (83). In alleviating depression, serious games can be classified into exergames (games that make patients exercise) or computerized cognitive behavioral therapy (CBT) games. A recent meta-analysis showed that involving in exergames or computerized CBT games led to significantly less severe depressive symptoms compared to no intervention, with no significant difference between exergames and conventional exercises (84).

Regardless of the medical conditions targeted, there is always a need for interdisciplinary collaborations to effectively harness the potential of digital health technologies (85).

### 2.2. Recent targets and trends in digital health technologies

There is a whole spectrum of AI/ML technology that can be applied in a clinical environment. One example is to use AI/ML to such as to screen electrocardiograms (ECGs) to identify abnormal heart rhythms and facilitate healthcare decision making (86).

It has been estimated that currently every individual has 6–7 mobile devices connected to the internet, rendering IoT and even the Internet of Medical Things (IoMT) more practical than before (87). For instance, IoMT sensors could be used to observe the behavior of a person at risk of early dementia by collecting patient data and comparing it with expected behavior according to the existing database (88, 89). Wearable sensors, sensors installed at home, and the wireless sensor networks altogether could form a comprehensive data collection inventory that monitors the disease progression of a dementia patient by noticing abnormality both at home and outside home (90). These examples illustrate the large amount of data that might be collected from the field sensors (edge level) and its potential transfer to the cloud (cloud level) for high performance computing tasks and data storage. The generalization of the 5G network may provide improved speed, reliability, energy efficiency, and mobility for such systems (91). However, many factors might still hinder the prompt response or safety of such systems, such as security issues, cloud space allocation, and internet speed. As such, it was proposed that some AI algorithms could be introduced in the edge level or in the fog level (between the edge and the cloud, where the local computers and servers gather data and perform local processing and storage), so as to compensate for the shortcomings of the cloud (92).

With more and more AI algorithms being implemented for healthcare use, it is important to plan for algorithmic stewardship, which monitors the ongoing clinical use and performance of AI algorithms and ensures they are safe to be used (93). Apart from the application side, the development side should also establish a reference standard for the design, execution, and reporting of AI-related studies such as those assessing the diagnostic accuracy of AI (94, 95). It should be noted that in 2022 there were nearly 150 clinical trials on FDA-regulated digital therapeutics so that it is timely to increase the transparency in their reporting (96).

One of the most recent trends in the COVID-19 pandemic era was the development of robotics in healthcare to minimize direct human contacts to lower the risk of COVID-19 transmission. Some notable directions are listed in Table 5.

**Table 5:** Examples of robot use in the COVID-19 pandemic era.

Robot type	Details
Delivery robot	Deliver foods and medicines to patients with COVID-19 in quarantine zone, or to customers in restaurants and older adults homes
Screening robot	Conduct swab tests for mass screening of the public regarding COVID-19, identify individuals with high temperature in the crowd
Surgery robot	Assist or perform surgeries in operation theaters
Disinfection robot	Clean and disinfect places in hospitals, restaurants and shopping malls
Public health robot	Promote health awareness, distribute masks and hand sanitizers
Social robot	Communicate with patients in quarantine, enable face calls with family members in other locations, serve as staff in the kiosk

The table is based on Wang and Wang (97) and Naik et al. (49).

### 2.3. Advocation of digital health technologies by the World Health Organization

**Figure 1:**
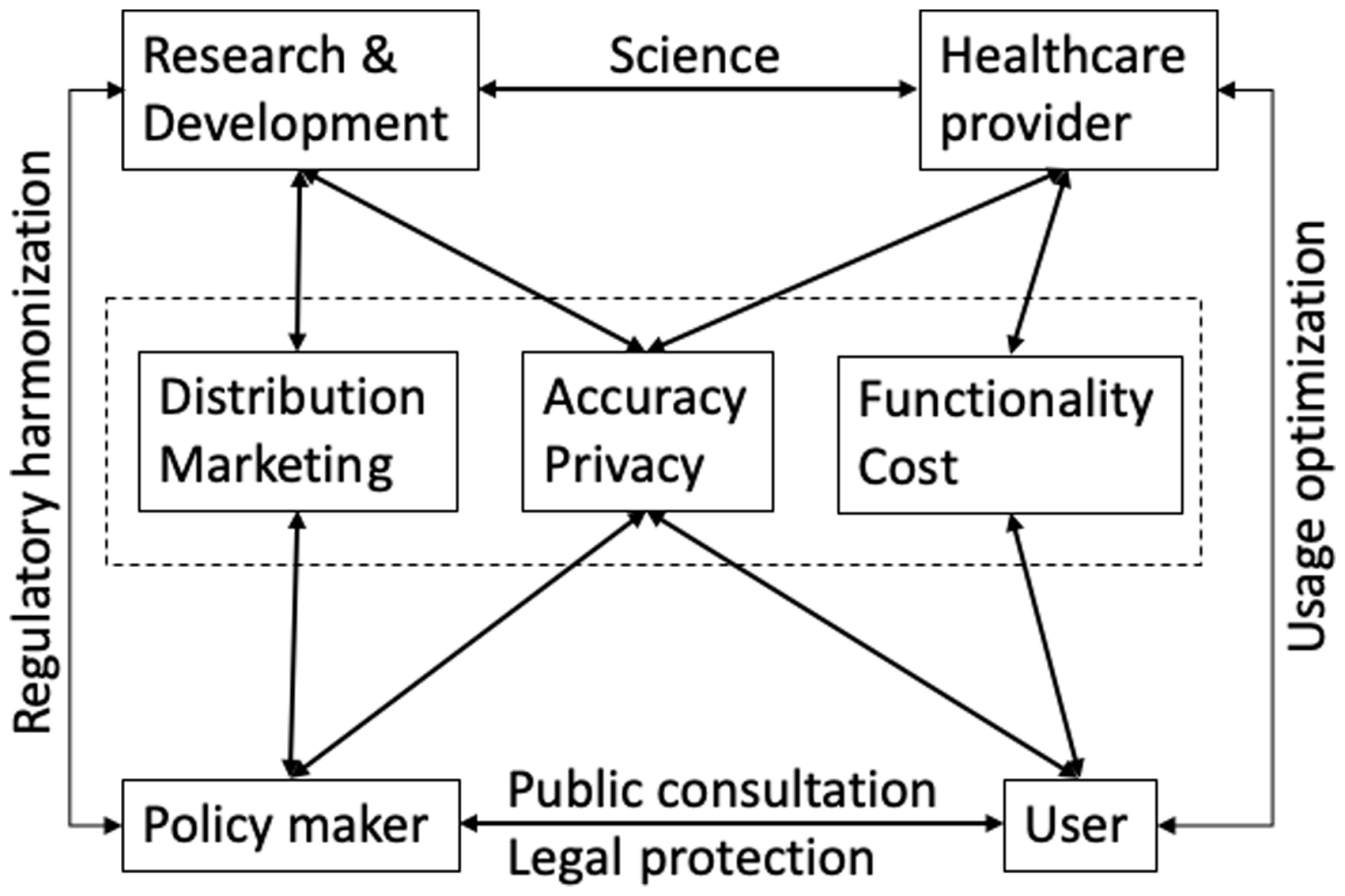
The integration of digital health technologies into the existing healthcare system requires input from various stakeholders. Partially based on Naik et al. (49).

With the fast growing number of digital health products such as AI-driven solutions and health apps, the role of regulatory bodies becomes even more paramount than before. A recent article summarized the regulatory approaches from nine countries on health app policy (98). In brief, some countries have already established their regulatory framework. For example, Singapore stipulated that apps must be approved by the Health Sciences Authority prior to their release for use, and are regulated by laws and non-legally binding guidelines. Meanwhile in the United States, apps that were classified as medical devices and of moderate or high risk should be approved and regulated by the Food and Drug Administration (FDA). Table 6 shows some examples of regulatory and ethical considerations related to the use of digital health technologies in different countries or regions. However, many health apps were not considered to have met the abovementioned criteria to be regulated. Taking into consideration that relevant regulatory frameworks are still to be fully established and the application of digital health technologies at international scale is complicated by the diversity of legislations and approaches by different countries, guidance by international health bodies such as the WHO can be of great value. Overall, regulatory frameworks and policies should prioritize patient safety, data privacy and security, interoperability, ethics, clinical validation, patient-centered approach, and regulatory harmonization. The integration of digital health technologies into the existing healthcare system requires input from various stakeholders (Figure 1).

**Table 6:** Examples of regulatory and ethical considerations related to the use of digital health technologies in different countries or regions.

Country/Region	Regulatory considerations	Ethical considerations	Reference
United States	FDA regulations apply to digital health technologies intended for medical use	Protect patient safety, privacy concerns on patient data, sharing regulatory duties with developers to balance between regulation and space for innovation	(99)
European Union	The CE marking is required for certain digital health technologies, and the General Data Protection Regulation (GDPR) applies to data privacy	Balance the protection of individual privacy and the promotion of growing European data economy	(100, 101)
Canada	Health Canada regulates digital health technologies that meet the definition of medical devices	Ensure patient safety in the use of digital health technologies, to reduce barriers to market entry, stimulate innovation, and encourage adherence	(102)
Australia	The Therapeutic Goods Administration (TGA) regulates digital health technologies that meet the definition of medical devices	Protect data privacy, and protect patient safety	(103)
China	The National Medical Products Administration (NMPA) regulates digital health technologies that meet the definition of medical devices	Foster patient safety and device reliability	(104)

The WHO has formed a Global Strategy on Digital Health aimed to bring together the global digital health community to get involved in the digital transformation of health (105). The WHO has also published nine recommendations on mHealth interventions (106). To broaden the reach of public health messages, the WHO has created a Health Alert Chatbot available through WhatsApp, Facebook Messenger, and Viber to provide information on safety measures for COVID-19, disease prevention, symptoms, and short-term and long-term effects (107). The WHO also released two mobile apps, the WHO Academy: COVID-19 Learning App and the WHO Info App, to provide up-to-date information for clinicians and patients/healthcare consumers, respectively (108). A physical 9-storey WHO Academy hub is currently under construction in Lyon, France. It is expected to be opened in 2024, and will offer spaces for in-person and distance learning, which include a health emergencies simulation center and customized distance and hybrid-learning classrooms (109).

### 2.4. Advanced digital health technologies in clinical trials

Digital technologies can facilitate clinical trials. A recent trial demonstrated real-time perspiration analysis with multiple simultaneous measurement of sweat metabolites (glucose and lactate) and electrolytes (sodium and potassium) with skin temperature (110). Plastic-based skin sensors were incorporated in a wristband or forehead patch, accompanied with an Android app (110). Meanwhile, another trial found that smartwatch and activity tracker data, together with self-reported symptoms and diagnostic testing results, had superior performance to detect COVID-19 infection among symptomatic individuals compared to considering symptoms alone (111). Popular smartwatches such as by Garmin and the Apple Watch were frequently involved in clinical trials. In one trial, the use of Garmin together with a behavioral feedback and goal-setting session and 5 telephone-delivered health coaching sessions significantly reduced both total sitting time and prolonged bouts of sitting among breast cancer survivors, compared to no intervention (112). Concurrently, a trial on the Apple Watch found that among participants who received notification of an irregular pulse, 34% had atrial fibrillation on subsequent ECG examination and 84% of notifications were concordant with atrial fibrillation, implying the usefulness of the app for early detection (113).

Meanwhile, some clinical trials showed that digital health technologies were not helpful. For instance, in a trial of 850 patients with heart failure, the positive effects of a 9-week program of hybrid comprehensive telerehabilitation did not increase the percentage of days alive and out of the hospital, and did not reduce mortality and hospitalization over a follow-up period of 14 to 26 months (114). An early trial of 3,230 patients with diabetes found that the addition of telehealth (remote exchange of data between patient and healthcare providers) reduced the mortality rate at 12 month follow-up, but did not improve the mean number of emergency admissions per patient after adjusting for baseline characteristics (115, 116).

### 2.5. GP and doctor training by digital health/AI/ML

**Figure 2:**
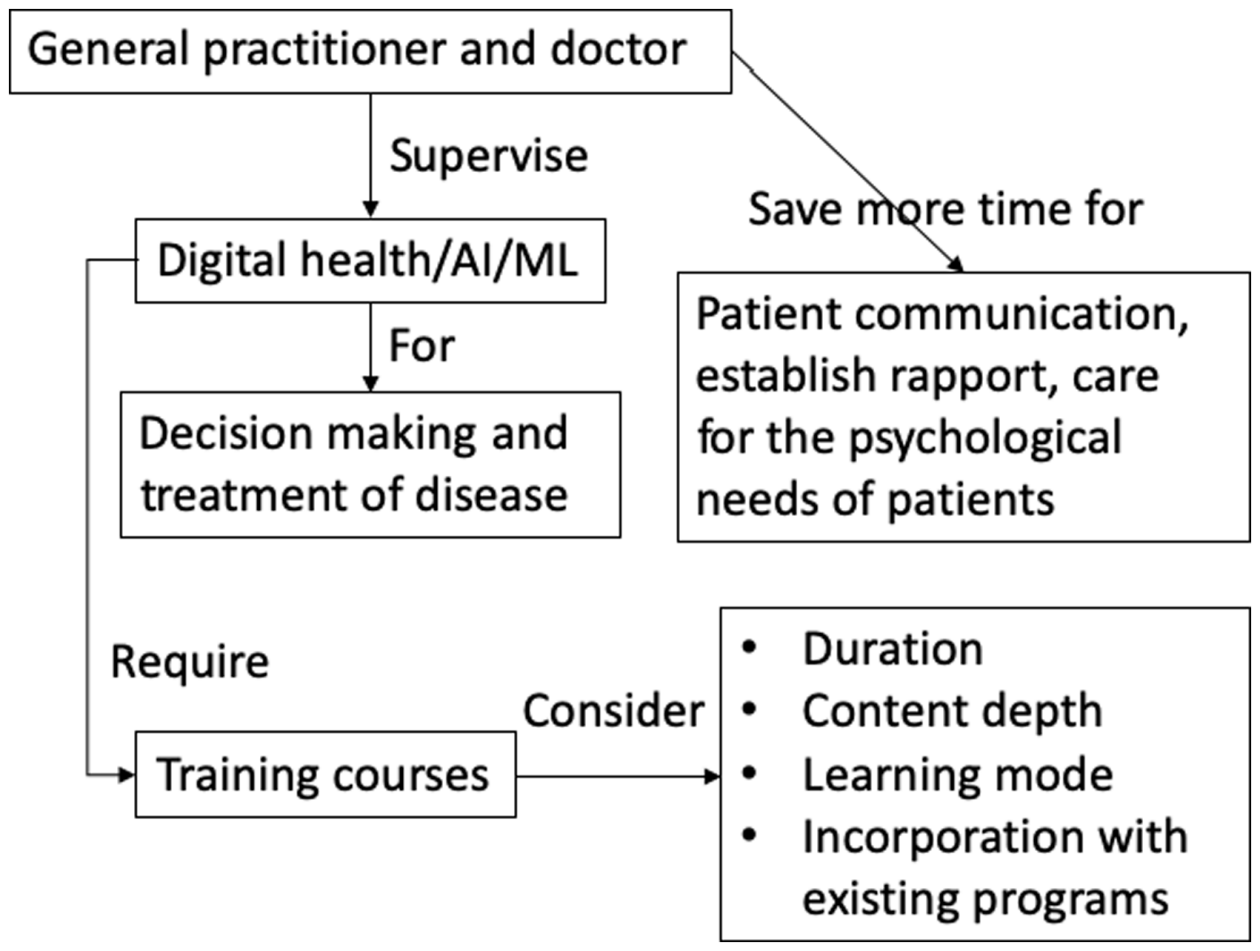
Schematic diagram of GP and doctor training by digital health/AI/ML.

Digital health technologies not only could benefit patients but also might enhance the training of GPs and healthcare professionals. For example, an e-learning environment could store videotaped trainees’ information-giving sessions, and enable feedback on the sessions from peers, communication experts and patients, so that ultimately the polished skills could be transferred to daily clinical practice (117). Some researchers even compared the combination of digital health technology and AI/ML with general automation technology in aviation, so that a future physician’s role would become a supervisor of patient healing by interacting with AI/ML or ensuring the accuracy of the decision-making by AI/ML (118). However, instead of being idle, the future physicians should be able to spend more time on the patient-doctor relationship, establish rapport, and care for the psychological/mental needs of the ill patients (118). On the other hand, GPs and healthcare professionals need to learn computer science skills to master AI/ML programs and fully utilize their potentials. There are many training programs on the market with diverse duration and content depth. From a recent report on 100 AI training programs for radiologists, most of the programs were found to be short, stand-alone sessions; focused on the basic concepts of AI; mainly covered medical, technical aspects but not managerial, legal and ethical topics; and offered in passive mode (no hands-on) (119). Perhaps future AI/ML education should start at the undergraduate level in healthcare education programs, so that the students could build up their AI/ML knowledge with medical knowledge in a more coherent way (120–122). Figure 2 is a schematic diagram that summarizes this section.

## 3. Conclusion

**Figure 3:**
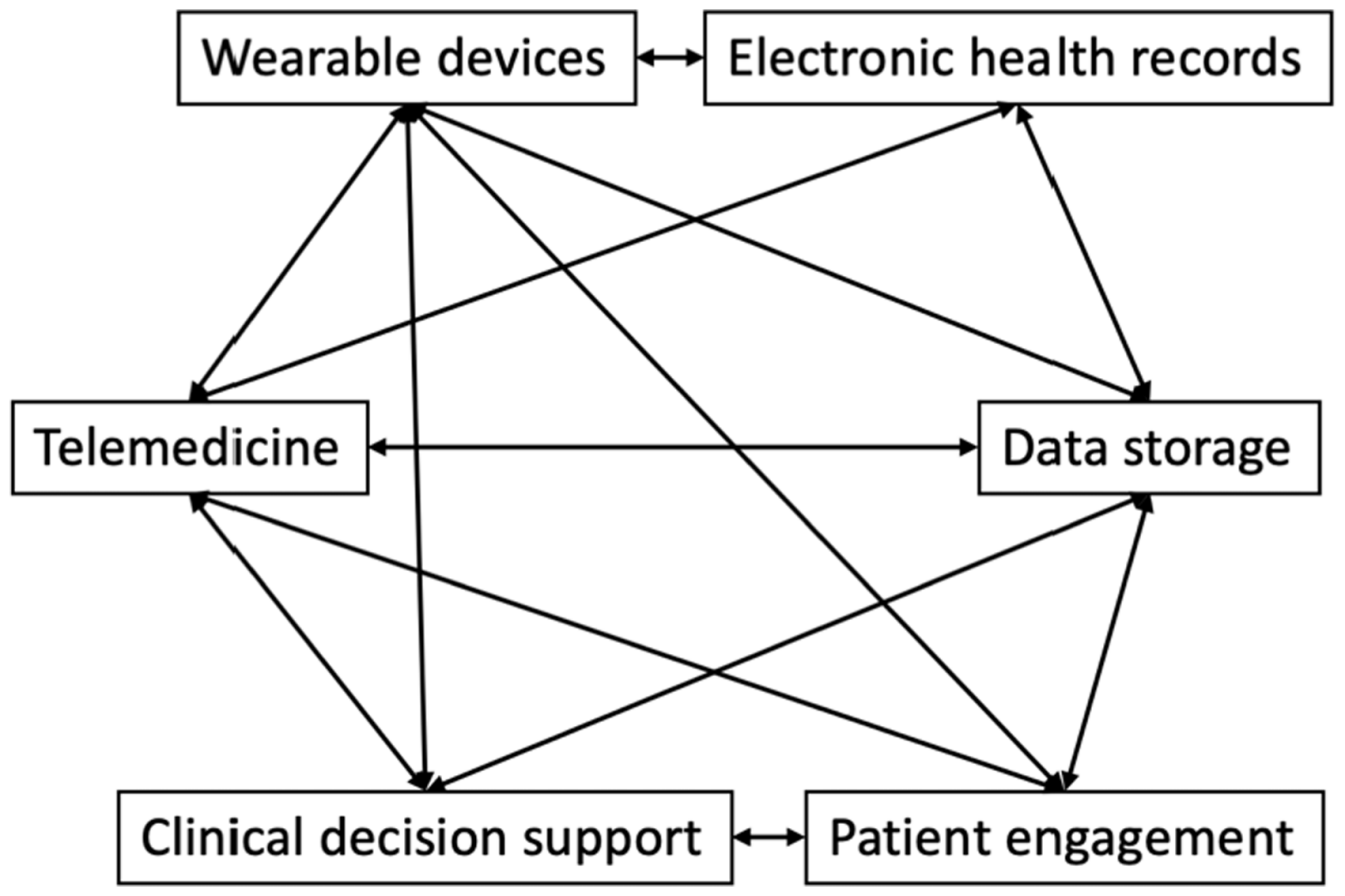
The interconnections between various components in digital health technologies.

**Figure 4:**
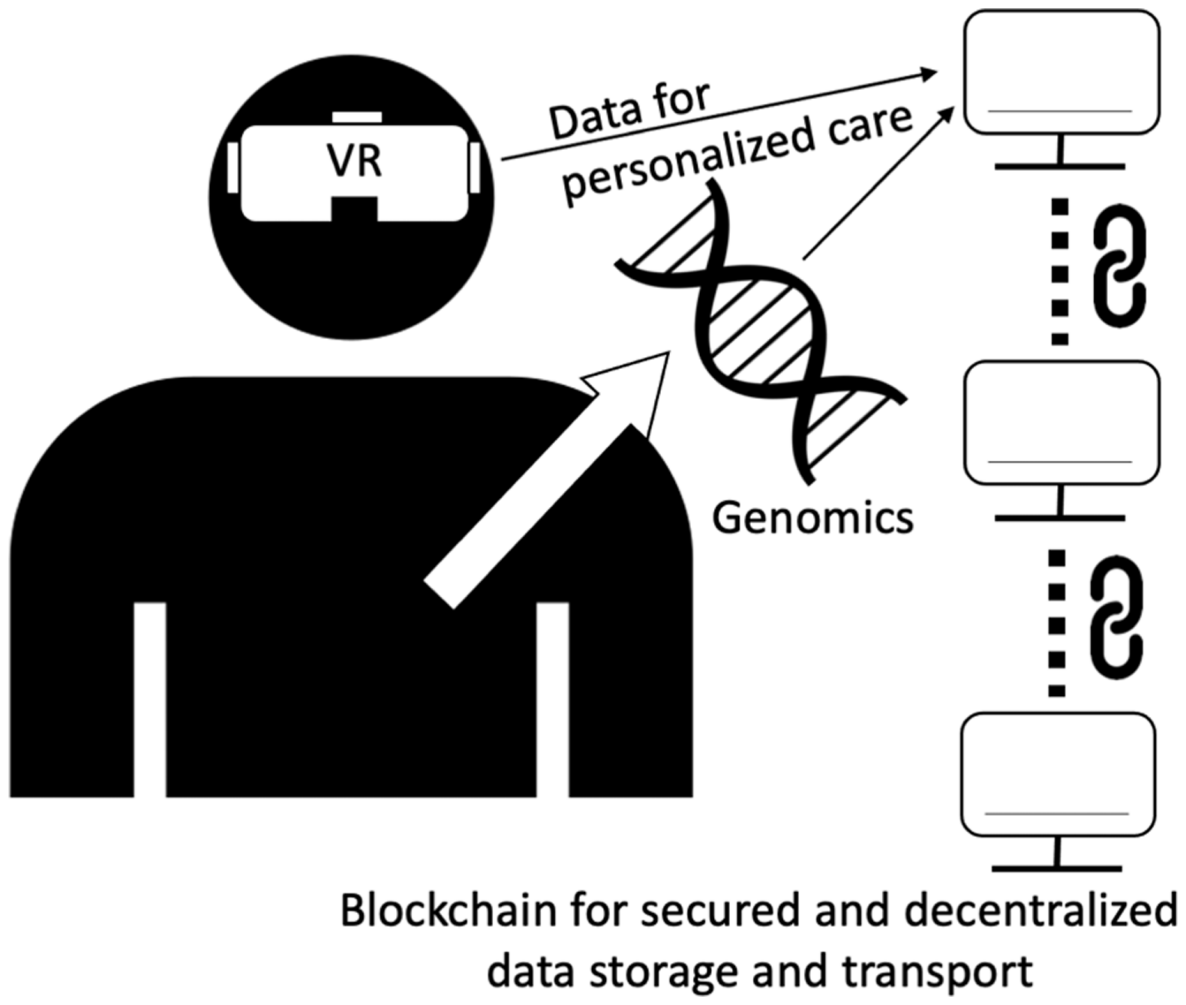
The future trend of digital health technologies with virtual reality, genomics, or blockchain.

This review has covered the current state of the scientific field of digital healthcare technologies, the promise of the technologies, the current limitations and challenges, and the potential for applications in different healthcare scenarios. A summary of the key features, advantages, limitations of different digital health technologies is presented in Table 7. The interconnections between these components are now illustrated in Figure 3. It is clear that the future of medicine and healthcare will involve increasing adoption of various kinds of digital technologies. Some, such as the use of cloud computing that incorporates AI/ML analytics are especially promising. Many routine aspects of the healthcare pathway will be automated (e.g., verified with bar codes/QR codes/wireless RFID to reduce manual errors), and the diagnostic and management aspects of clinical care will likely be more personalized (with consideration of multi-omics data and real-time health surveillance data with wearable sensors). The implementation of digital health technology in healthcare will result in different clinical workflows and would need to implement more Quality Control/Standard Operating Procedures to maintain the integrity and consistency of digital apps over time and over the total of costs of ownership. Along this line, algorithmic stewardship should be implemented with the consideration of total ownership perspectives, and validation should be applied more than once, as demographics and care procedures change over time. Importantly, innovative approaches that would accelerate the research, translation and utilization of digital health technologies are critically needed. Moreover, future will witness more digital health workflows going beyond hospital walls and inpatient care, and this would also require proper training of caregivers and patients in using digital health apps. Certainly, digital health comes with challenges. Various stakeholders in the healthcare sector may be hesitant and concerned with the changes. Some changes and digital health implementations may be reverted or stopped once the health and manpower concerns associated with COVID-19 are over. On the other hand, the use of semi-or fully automated robots to perform various tasks such as food and medication delivery to patients and disinfection of hospital seem to have a good reception and may continue in the future. Healthcare providers may have many concerns, such as being replaced by digital health technologies, changes in their duties, and being unable to adapt to the new working duties and environment. Patients may also fear of having worse quality of care and less direct communication with care providers. However, advances in digital health technologies seem to be unavoidable and their usage will spread to less resourceful countries once they are produced and implemented in mass scale with reduced costs. Virtual reality, genomics, and blockchain may play important roles in the future of digital health technologies and require more research in clinical settings with different patient groups (Figure 4).

**Table 7:** Summary of key features, advantages, limitations of different digital health technologies discussed in this review.

Technology	Key features	Advantages	Limitations
Telemedicine	Video consultations with healthcare providers	↑ Access to professional care, ↑ convenience, ↓ travel time and costs	Inability to conduct physical exams, potential for technical difficulties especially on the patient side
Wearables	Devices worn on the body to track health metrics	Continuous data collection and monitoring, ↑ patient awareness	Questionable accuracy in some metrics/devices
AI-based diagnostics	Use of artificial intelligence for data analysis and diagnosis	↑ Efficiency, ↓ costs, No human error	Current AI models may have limited ability to handle complex cases, potential errors/bugs in algorithms
Mobile health apps (mHealth)	Smartphone apps for tracking health metrics and managing conditions	↑ Patient engagement and self-management, ↑ access to health information	Limited accuracy and reliability in some metrics, potential for data privacy concerns

Readers can refer to the respective parts of the main text for specific literature references.

## Author contributions

AY and AA conceived, designed, and coordinated the writing of the whole manuscript. All the authors contributed to critically revise and approve the final version of this manuscript.
